# PLA/PBS Biocomposites for 3D FDM Manufacturing: Effect of Hemp Shive Content and Process Parameters on Printing Quality and Performances

**DOI:** 10.3390/polym17172280

**Published:** 2025-08-23

**Authors:** Emilia Garofalo, Luciano Di Maio, Loredana Incarnato

**Affiliations:** Department of Industrial Engineering, University of Salerno, Via Giovanni Paolo II, 84084 Fisciano, SA, Italy; ldimaio@unisa.it (L.D.M.); lincarnato@unisa.it (L.I.)

**Keywords:** 3D-FDM printing, PLA/PBS blend, hemp shive, viscoelastic properties, mechanical properties

## Abstract

This study investigates the processability—via Fused Deposition Modeling (FDM) 3D printing—and mechanical performance of biocomposites based on polylactic acid (PLA), polybutylene succinate (PBS), and their 50/50 wt% blend, each reinforced with hemp shive at 3 and 5 wt%. Blending PLA with PBS represents a straightforward and encouraging strategy to enhance both the printability and mechanical properties of the individual resins, expanding the range of their potential applications. The addition of hemp shive—a by-product of hemp processing—not only enhances the biodegradability of the composites but also improves their thermo-mechanical performance, as well as aligning with circular economy principles. The rheological characterization, performed on all the systems, evidenced that the PLA/PBS blend possesses viscoelastic properties well suited for FDM, enabling smooth extrusion through the nozzle, good shape stability after deposition, and effective interlayer adhesion. Moreover, the constrain effect of hemp shives within the polymer matrix reduced the extrudate swell, a key factor affecting the dimensional accuracy of the printed parts. Optimal processing conditions were identified at a nozzle temperature of 190 °C and a printing speed of 70 mm/s, providing a favorable compromise between print quality, final performances and production efficiency. From a mechanical perspective, the PLA/PBS blend exhibited an 8.6-fold increase in elongation at break compared to neat PLA, and its corresponding composite showed a ductility nearly three times higher than the PLA-based counterpart’s. In conclusion, the findings of this study provide new insights into the interplay between material formulation, rheological behavior and printing conditions, supporting the development of sustainable, hemp-reinforced biocomposites for additive manufacturing applications.

## 1. Introduction

The environmental impact of traditional plastics has prompted research to focus on more sustainable materials, such as biodegradable polymers. These materials are also of great interest in cutting-edge technological fields, including 3D printing.

Additive manufacturing (AM) involves the fabrication of objects by depositing successive layers of material based on a CAD (Computer-Aided Design) model. Among the different AM technologies, Fused Deposition Modeling (FDM) is the most widespread [1]. In this process, a thermoplastic filament is heated to its melting point and extruded through a nozzle. FDM not only offers low production costs, but also enables the creation of complex geometries while minimizing material waste and reducing the production of scraps [1].

One of the most commonly used materials in FDM is PLA (polylactic acid), a biodegradable aliphatic polyester. PLA can be processed at lower temperatures than traditional polymers, like ABS and polyamides, making it a more sustainable option. Because of its high strength, high modulus, biodegradability, and biocompatibility, PLA is widely used in drug delivery systems, implantable scaffolds, wound care, sutures, and so on [2]. However, PLA’s inherent brittleness has limited its broader application. To tackle this issue, several studies [3,4,5,6,7,8] have shown that blending PLA with tougher polymers can improve its elasticity.

PBS (polybutylene succinate), a semi-crystalline aliphatic polyester, offers complementary properties to PLA. PBS features excellent thermal stability, biodegradability, a low melting point (below 120 °C), and high ductility [4,6]. Nevertheless, its use in FDM 3D printing is still under investigation [9,10,11,12] due to its high crystallization rate, which can cause significant shrinkage during printing, leading to defective prints. Therefore, PBS requires modification to be suitably processed via FDM, and blending it with PLA is a straightforward and encouraging approach [9,11,12]. The combination of PLA and PBS offers a good balance of strength and ductility [4,13,14]; therefore, PLA/PBS blends may serve as promising biodegradable and biocompatible materials for additive manufacturing, with potential applications across various fields—from bioengineering and medical devices to wearable smart technologies (such as smartwatches, fitness trackers, and augmented reality glasses). Notably, some studies have reported that blends with more than 50 PBS by weight become unprintable [11], and that the optimal printing temperature for PLA/PBS blends is around 250 °C [12].

In a sustainability-driven context, repurposing materials traditionally viewed as waste—such as lignocellulosic by-products from agriculture—plays a key role in promoting both environmental preservation and global economic growth. This strategy aligns with the principles of the circular economy by maximizing the value extracted from natural resources. Among these materials, lignocellulose-based biocomposites are receiving increasing attention [15,16,17]. Yet, there are still few studies addressing the use of natural-origin fillers in matrices like PLA or PLA/PBS blends for additive manufacturing [18,19]. In particular, hemp shives, a low-value by-product from hemp fiber processing, can be milled into fine particles and used as reinforcement in polymer composites [20,21,22,23,24,25,26,27]. Beyond their biodegradability and low environmental impact, hemp shives have been shown to accelerate the composting process of biocomposites compared to unfilled polymers. In fact, due to their hygroscopic nature, hemp shives facilitate the absorption of moisture into the polymer matrix, initiating hydrolysis. The latter is the rate-limiting step in the degradation of bio-polyesters, which otherwise degrade very slowly under ambient temperature and humidity [21,28,29]. Moreover, hemp-based composites demonstrate enhanced mechanical performance, including increased tensile modulus at room temperature and improved heat deflection temperature [30].

This study explores the use of hemp powder—obtained by grinding hemp shive—as a natural filler in PLA, PBS, and a PLA/PBS blend at 50/50 by weight. The aim is to assess the processability of these biocomposites by FDM 3D printing, highlighting the correlations among the systems’ rheological behavior, their printability and final mechanical performances.

To be considered printable via FDM, a material must fulfill three essential criteria [31,32,33,34,35,36]. First, it must exhibit a melt flow behavior to allow for smooth and continuous extrusion through the printer’s nozzle. Second, once deposited, the extruded filament must possess adequate viscoelastic properties to retain its shape and avoid excessive spreading or deformation on the print bed. Finally, during the cooling phase, the printed layers must maintain dimensional stability to preserve the intended geometry; moreover, the polymer chains must inter-diffuse between adjacent layers to ensure good interfacial adhesion, which is critical for the mechanical integrity and compactness of the final printed object. These aspects were investigated through a rheological characterization performed with both the neat materials and the hemp-reinforced composites, focusing on their shear-thinning behavior, viscoelasticity properties and die-swell phenomenon. Following the rheological analysis, filaments, with a nominal diameter of 2.85 mm, were produced for 3D printing.

The second phase of this experimental work focused on the effect of printing parameters—particularly nozzle temperature and extrusion speed—on the quality appearance and mechanical performance of the printed samples. Surface and dimensional accuracy were assessed using an optical microscope. In addition, the flexural and tensile properties of the printed specimens were measured as a function of printing temperature and filler content.

## 2. Materials and Methods

### 2.1. Materials and Composites Production

In this study, a bio-based and biodegradable grade of polybutylene succinate (PBS) (Bio PBS™ FZ91, by Mitsubishi Chemical, Düsseldorf, Germany) and a polylactic acid (PLA) grade containing 1.5 wt% of the D-isomer (Ingeo™ Biopolymer 4032D, by NatureWorks, Naarden, The Netherlands) were employed. Their key properties are summarized in Table 1. The hemp shive employed in the composite formulations was mechanically milled and subsequently sieved to isolate the particle fraction with sizes in the range of 125 ÷ 180 μm.

Composites based on PLA, PBS, and a PLA/PBS blend (containing 50 wt% of each polymer) were prepared with 3 wt% and 5 wt% of hemp shive via melt compounding using a co-rotating, intermeshing twin-screw extruder (Dr. Collin GmbH Maitenbeth, Germany —ZK 25-48D; screw diameter = 25 mm, L/D = 42). Prior to extrusion, the neat polymer pellets and composite mixtures were dried in a vacuum oven at 80 °C for 18 h. Extrusion was performed at a screw speed of 200 rpm, with the temperature profile adjusted based on the polymer matrix used, as detailed in Table 2. The extruded strands were cooled in a water bath and subsequently pelletized.

### 2.2. Filament Production and FDM Printing

Filaments for 3D printing were successfully produced from both the neat resins and the hemp-shive-filled composites without any processing issues. Filament extrusion was carried out using a benchtop single-screw extruder (Filament Extruder NEXT 1.0, by 3DEVO, Utrecht, The Netherlands) equipped with a four-zone heating system, cooling fans for filament solidification and an optical sensor for automatic diameter control. The extrusion was conducted at 5 rpm, while the temperature profile and cooling rate were optimized according to the specific material processed (as detailed in Table 3) in order to obtain filaments with dimensional consistency and a target diameter of 2.85 mm, compatible with the 3D printer used.

Three-dimensional printing tests were conducted using the produced filaments to fabricate simple rectangular specimens with dimensions of 50.8 × 12.7 × 1.5 mm and 90 × 12.7 × 1.0 mm (Length × Width × Thickness), intended for the flexural and tensile mechanical characterization of the different systems, respectively. Printing was carried out using an Ultimaker 3 printer (Ultimaker, Utrecht, The Netherlands) equipped with a 0.4 mm diameter nozzle. Three different nozzle temperatures were tested (190 °C, 220 °C, and 250 °C), while the build plate temperature was kept constant at 60 °C. Two printing speeds were tested (50 mm/s and 70 mm/s), while the layer height was set to 0.1 mm. A zig-zag pattern was applied for both the top/bottom layers and the internal infill, which was set at 100% density.

### 2.3. Characterization Techniques

Thermogravimetric analysis (TGA) was performed using a TGA/DSC+3 instrument (Mettler-Toledo International Inc., Greifensee, Switzerland) in accordance with ASTM E1131–03. The measurements were conducted under a nitrogen atmosphere from 25 °C to 1100 °C, at a heating rate of 10 °C/min.

Fourier-transform infrared spectroscopy (FT-IR) analyses were carried out using a Nicolet Apex spectrometer (Thermo Scientific, Waltham, MA, USA) equipped with a SmartPerformer accessory for attenuated total reflectance (ATR) measurements. Each spectrum was acquired by averaging 64 scans over a wavenumber range of 4000–650 cm^−1^ with a resolution of 2 cm^−1^.

Dynamic shear rheological measurements were performed using an ARES rotational rheometer (Rheometric Scientific, New Castle, DE, USA) equipped with parallel plates of 25 mm diameter. Tests were conducted at 190 °C under a nitrogen atmosphere, over an angular frequency range of 0.1–100 rad/s and setting a gap of 1 mm between parallel plates. A strain amplitude of 5% was selected to ensure operation within the linear viscoelastic regime, as verified by preliminary strain sweep tests.

To measure die-swell, all materials were extruded through a 0.4 mm nozzle at a constant feed rate, equal to the Ultimaker 3 printer’s filament loading speed.

The printed specimens, as well as the filaments extruded by the printer nozzle, were observed with an Axioskope 40 Amicroscope (Carl Zeiss Vision, München-Hallbergmoos, Germany).

Tensile mechanical tests were conducted in accordance with ASTM D882 using a CMT4000 Series universal testing machine (SANS, Shenzhen, China). A crosshead speed of 30 mm/min was applied and a 1 kN load cell was employed for the tensile tests.

Flexural properties were evaluated following the ASTM D790 standard, using the same CMT4000 Series testing machine equipped with three-point bending fixtures and a 1 kN load cell. The crosshead speed was set to 7.5 mm/min. For each system analyzed, the reported mechanical data represent the average values obtained from five individual specimens.

## 3. Results and Discussion

To assess the printability by the FDM 3D process of hemp shive composites based on PLA, PBS, and their 50/50 wt% blend, their rheological characterization was carried out. The analysis focused on shear-thinning behavior, viscoelastic properties, and the die-swell phenomenon—key factors influencing the materials’ suitability for FDM processing. However, some preliminary characterizations of the selected materials were performed to support a comprehensive understanding of the obtained results.

Subsequently, the influence of printing parameters—specifically nozzle temperature and printing speed—on the quality appearance and mechanical performance of the printed samples was investigated. Surface finish and dimensional accuracy were evaluated through optical microscopy, while flexural and tensile properties were analyzed in relation to both the printing temperature and filler content of the specimens.

### 3.1. Preliminary Characterization of Hemp Shive and Its Composites

**Figure 1:**
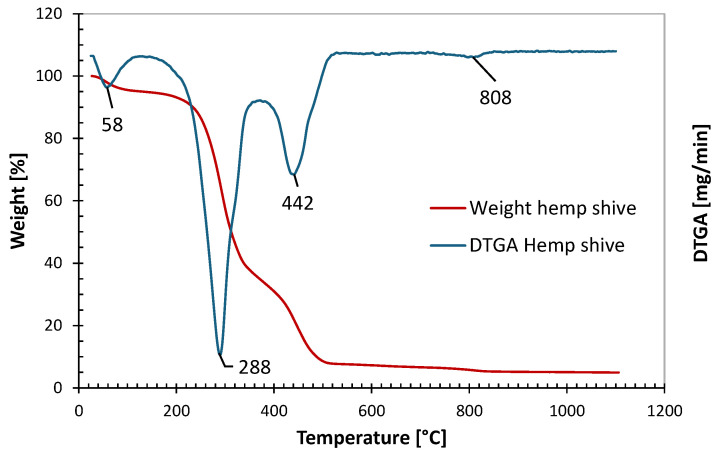
TGA and DTGA plots of hemp shive.

Lignocellulosic fillers are generally susceptible to thermal degradation when exposed to high temperatures. Therefore, in order to determine the upper temperature limit for processing hemp shive composites via 3D printing, a thermogravimetric analysis (TGA) was performed on the filler. Figure 1 shows the weight loss curve of the hemp shive and the corresponding derivative thermogravimetric analysis (DTGA) as a function of temperature.

Hemp shive is a lignocellulosic biomass primarily composed of cellulose, hemicellulose, and lignin, which decompose within characteristic temperature ranges, overlapping each other in some extent [37,38,39,40]. The initial mass loss observed between 30 and 120 °C can be attributed to the evaporation of physically absorbed moisture, accounting for approximately 5 wt% of the hemp shive. Although present in minor amounts, pectin also plays a significant role and begins to degrade earlier than the major components of hemp shive—around 180 to 200 °C—due to its low molecular weight and thermally unstable chains. Among the primary constituents, hemicellulose degrades first (200–300 °C), followed by cellulose (300–380 °C) with sharper mass loss, while lignin decomposes slowly over a broad range (250–600 °C) due to its complex structure. Above approximately 800 °C, an unburned mass remains (about 5%). Summing up, TGA analysis showed that hemp shive remains thermally stable up to around 200 °C, except for the early degradation of pectin.

An additional critical factor in evaluating the processability of a polymer blend and/or a composite—regardless of the specific processing technique—is the degree of compatibility between its constituents. This aspect plays a critical role in governing the flow behavior of the blend or composite during processing, as well as in determining its mechanical performance once solidified.

**Figure 2:**
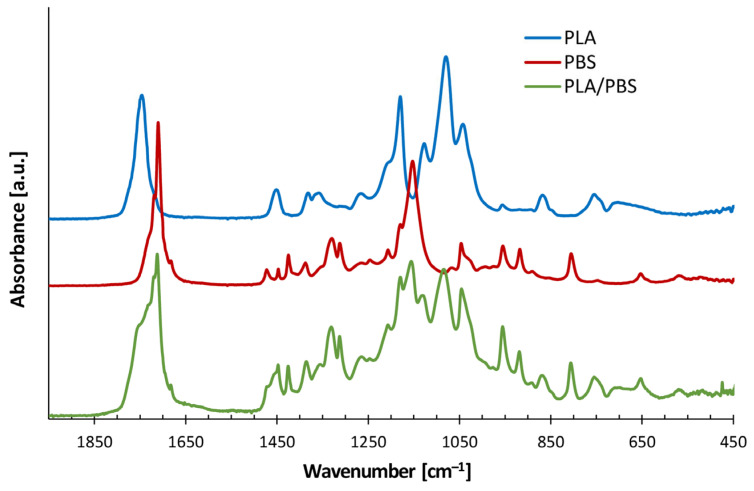
Comparison of ATR-FTIR spectra of PLA, PBS, and the PLA/PBS (50/50) blend.

To assess the compatibility of the selected system, FTIR spectroscopy was performed on neat PLA and PBS resins, as well as on their 50/50 wt% blend. A comparison of the spectra, shown in Figure 2, revealed that the blend retained the characteristic absorption bands of both neat polymers [41,42], without significant shifts in their wavenumbers (Table 4).

This suggested a lack of specific interactions between PLA and PBS phases, thereby confirming their immiscibility, in agreement with literature data [3,7,8,14].

**Figure 3:**
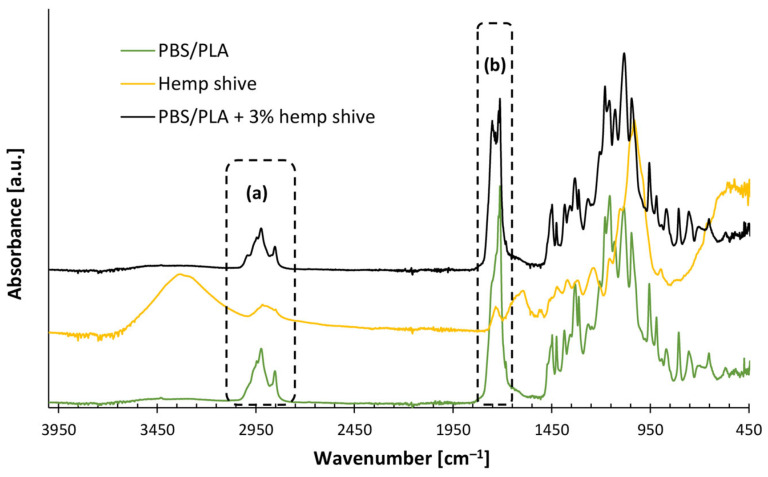

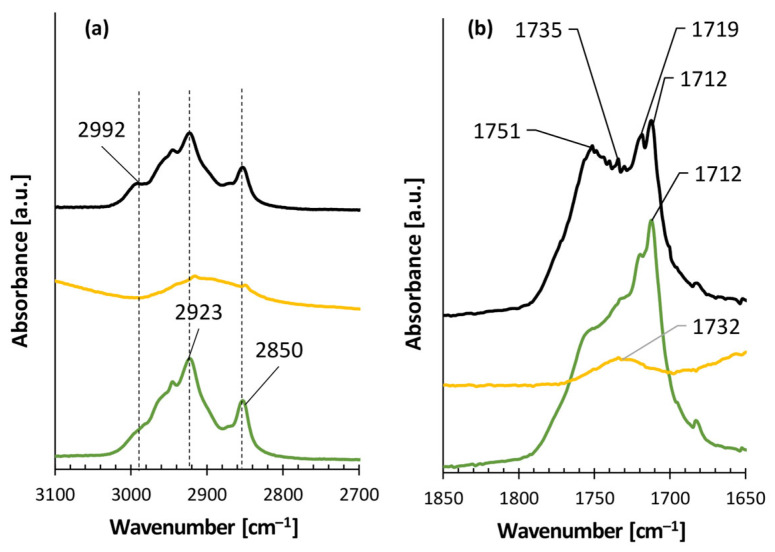
Comparison of ATR-FTIR spectra of hemp shive, neat PLA/PBS (50/50 wt%) blend and PLA/PBS with 3% hemp shive; (**a**) peaks details in the wavenumber region 3100 ÷ 2700 cm^−1^; (**b**) peaks details in the wavenumber region 1850 ÷ 1650 cm^−1^.

However, the incorporation of hemp shive into the PLA/PBS blend via melt compounding enhanced the interfacial compatibility between the two polymer phases. As shown in Figure 3, the FTIR spectrum of the composite displays two additional bands at 1735 and 1751 cm^−1^, corresponding to ester carbonyl stretching vibrations. These peaks—absent in both the neat blend and raw hemp shive (Table 4)—indicate that esterification reactions occurred. This is likely due to fumaric acid moieties along the PBS backbone [43,44], which can react (at temperatures above 180 °C) with hydroxyl groups (–OH) of hemp shive, forming covalent ester bonds and, thus, improving the adhesion among PLA, PBS, and the lignocellulosic filler.

### 3.2. Rheological Behavior of the Neat Systems and Hemp-Shive-Based Composites

To evaluate the flow behavior of the materials during printing, dynamic rheological measurements were carried out on the neat resins (PLA and PBS), their PLA/PBS blend, and the corresponding composite systems containing 3% and 5% by weight of hemp shive. In particular, the rheological characterization was carried out at 190 °C, as this is the typical printing temperature for PLA, which serves as the benchmark in this study. Additionally, TGA analysis (Figure 1) revealed that significant thermal degradation of hemp shives begins above 200 °C.

**Figure 4:**
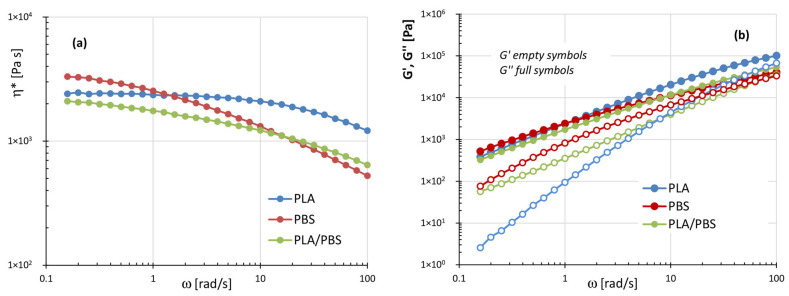
(**a**) Plots of the complex viscosity ƞ*; (**b**) storage modulus G′ and loss moduli G″ as a function of frequency for the systems: PLA, PBS, and PLA/PBS (50/50 wt%).

Figure 4a,b compares the plots of complex viscosity (η*), storage modulus (G′), and loss modulus (G″) for the unfilled systems: PLA, PBS, and the PLA/PBS blend.

The complex viscosity curve of PLA exhibits a relatively broad Newtonian plateau, followed by a moderately shear-thinning region. In contrast, PBS shows a pronounced decrease in η* with increasing angular frequency (ω) across the entire frequency range analyzed. However, at frequencies below 1 rad/s, PBS displays higher complex viscosity values than PLA. The PLA/PBS blend exhibits lower viscosity values than both neat polymers up to 10 rad/s, while at higher frequencies, its viscosity falls between those of PLA and PBS (Figure 4a). As shown in Figure 4b, the storage modulus (G′) of PLA/PBS blend closely follows that of PBS, with neat PBS exhibiting the highest G′ values across the entire frequency range. Moreover, both PBS and the blend display a more pronounced elastic behavior than PLA in the low-frequency region.

These results provide useful insights for a preliminary assessment of the printability of PLA, PBS, and their blend via FDM technology. Specifically, a pronounced shear-thinning behavior is desirable to ensure good processability during both the extrusion and deposition stages. In fact, low viscosity inside the nozzle reduces melt pressure, allowing for lower processing temperatures, while moderately high viscosity at the nozzle exit prevents issues such as material dripping or poor layer definition [32,33,34,35,36,45]. On the other hand, during the deposition phase, the interlayer cohesion—which directly influences the final mechanical properties of the printed object—is primarily governed by the diffusion [46] and relaxation of polymer chains across adjacent layers. In particular, enhanced interfacial diffusion is favored in systems exhibiting predominantly viscous responses at the deposition temperature [32,33,34,35,36].

Thus, PBS is expected to exhibit favorable flow characteristics for extrusion through the printer nozzle, along with effective shape retention upon exiting the die. This behavior is mainly attributed to its marked shear-thinning properties. Nevertheless, its higher elastic component, compared to PLA, may introduce some drawbacks, such as an increased tendency for die-swell at the nozzle exit (as highlighted in the following) and reduced interlayer adhesion during deposition. However, some studies in the literature [12,45] evidenced that the main issue, affecting PBS printability, does not stem from its rheological behavior but from its rapid crystallization kinetics. In fact, if crystallization occurs too rapidly, it can lead to pronounced warping and shrinkage phenomena, resulting, in some cases, in the detachment of the printed material from the build platform, ultimately causing the failure of the printing process.

On the contrary, PLA is characterized by a more dominant viscous behavior, which is expected to promote improved interlayer diffusion and, consequently, enhanced cohesion in the printed structure. With regard to the PLA/PBS blend, the equal proportion of the two aliphatic polyesters results in a rheological response that combines the advantages of both components—exhibiting a more pronounced shear-thinning behavior than neat PLA and a more viscous character than neat PBS—potentially offering a favorable compromise for FDM processing.

**Figure 5:**
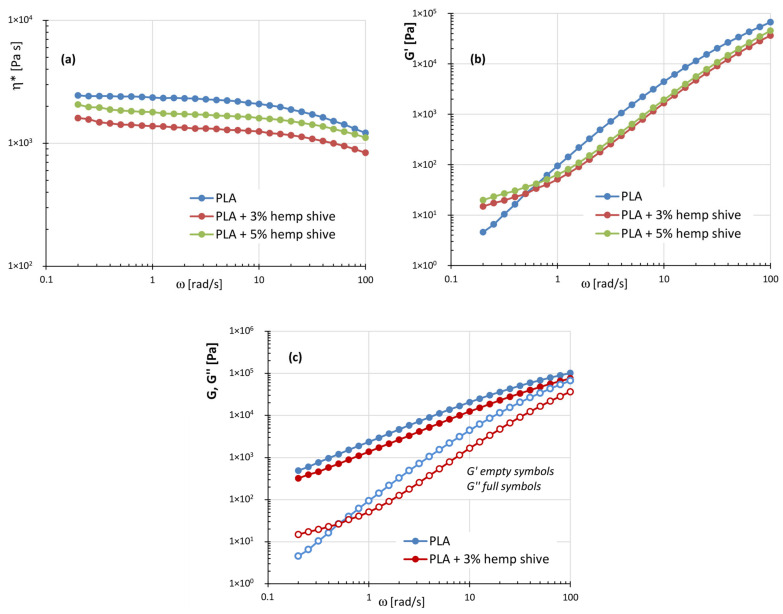
(**a**) Plots of the complex viscosity ƞ*; (**b**) storage modulus G′; (**c**) storage and loss moduli as a function of frequency for the systems: PLA, PLA with 3% and 5% of hemp shive.

Figure 5a–c presents the plots of complex viscosity, storage modulus, and loss modulus for PLA-based composites.

The complex viscosity curves (Figure 5a) for neat PLA and its composites exhibit similar overall trends. In particular, the filled systems display a Newtonian plateau at intermediate frequencies and a moderately shear-thinning behavior at higher angular frequencies, differing from the neat matrix essentially by the presence of a slight yield stress-like behavior at low ω. Moreover, across the entire frequency range analyzed, neat PLA exhibits higher complex viscosity than both the 3% and 5% hemp-filled composites. As supported by the thermogravimetric data in Figure 1, the filler undergoes partial degradation at the processing temperatures used during melt compounding with the polymer matrix (Section 2.1). In particular, according with the findings reported by Coppola et al. [47], the reduction in viscosity observed for the composites can be likely attributed to the thermal degradation of pectin, which therefore acts as a natural plasticizer inside the materials. Additionally, the lower viscosity of the composites compared to neat PLA can be partly attributed to the alignment of the anisotropic filler under flow.

The same behavior is observed for the storage modulus (Figure 5b), with the filled samples generally showing lower G′ values compared to the neat matrix, except in the low-frequency range. In this region, the composites also show a weaker dependence of G′ on frequency, suggesting a “pseudo solid-like” behavior. This feature, together with the observed yield stress, can be attributed to the development of a filler-induced network at low frequencies. Such structuring effect contributes to enhanced shape stability of the extrudate at the nozzle exit—where shear rates rapidly decrease—and during the subsequent deposition and solidification stages [32].

**Figure 6:**
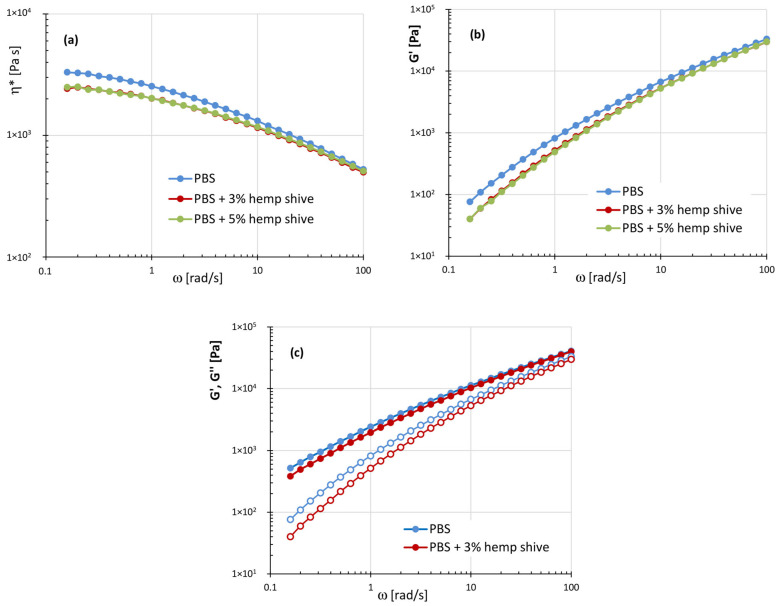
(**a**) Plots of the complex viscosity ƞ*; (**b**) storage modulus G′; (**c**) storage and loss moduli as a function of frequency for the systems: PBS, PBS with 3% and 5% of hemp shive.

Figure 6a–c shows the viscoelastic properties of the PBS-based systems: neat PBS, PBS with 3 and 5 wt% of hemp shive.

As observed for PLA-based systems, the addition of hemp filler within the PBS matrix results in a reduction in the viscoelastic parameters compared to the neat polymer, with the most significant variations occurring at low frequencies. Moreover, the plots of the composites essentially overlap across the entire frequency range examined, indicating comparable rheological behavior despite the different filler contents. The absence of a storage modulus plateau at low frequencies in the PBS/hemp shive composites can be reasonably attributed to weaker interfacial interactions between PBS and the filler, likely due to PBS’s lower polarity compared to PLA.

**Figure 7:**
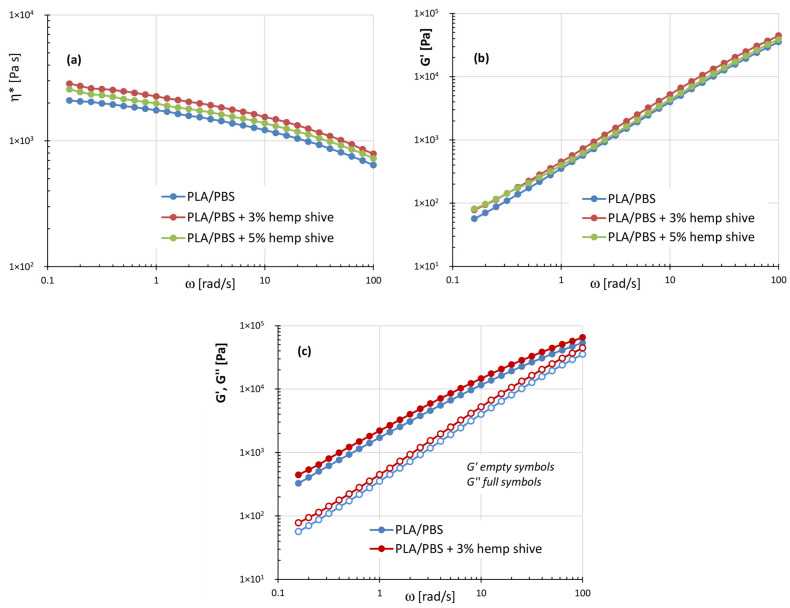
(**a**) Plots of the complex viscosity ƞ*; (**b**) storage modulus G′; (**c**) storage and loss moduli as a function of frequency for the systems: PLA/PBS, PLA/PBS (50/50 wt%) with 3% and 5% of hemp shive.

Figure 7a–c compares the complex viscosity and the storage and loss moduli of PLA/PBS blend and its corresponding hemp-filled composites.

Comparably to PLA- and PBS-based composites, the presence of hemp shive inside the PLA/PBS blend leads to a slight but consistent increase in the rheological parameters compared to the neat matrix. This behavior can reasonably be attributed to the compatibilizing effect of the filler in the blend, likely promoted by esterification reactions between the hydroxyl groups of hemp shive and the carboxylic acid groups of polymer phases, as confirmed by FTIR analysis (Figure 3). Due to the improved interaction, the PLA/PBS-blend-based composites also exhibit a slight reduction in the slope of the storage modulus curves, at low frequencies, compared to the neat matrix.

**Figure A1:**
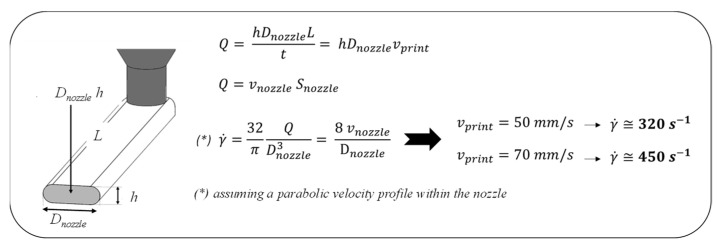
Procedure for estimating shear rates within the nozzle based on the selected printing parameters.

Although the results of dynamic rheological tests have provided valuable insights into the printability of the systems under investigation, a more comprehensive understanding of the material’s flowability through the printer nozzle can be achieved by determining their viscosity values at shear rates that realistically occur within the nozzle during printing. To this purpose, an approximate estimation of the shear rates, corresponding to the selected printing parameters, was conducted following a procedure reported in the literature [11] and described in detail in the Appendix A (Figure A1).

**Figure A2:**
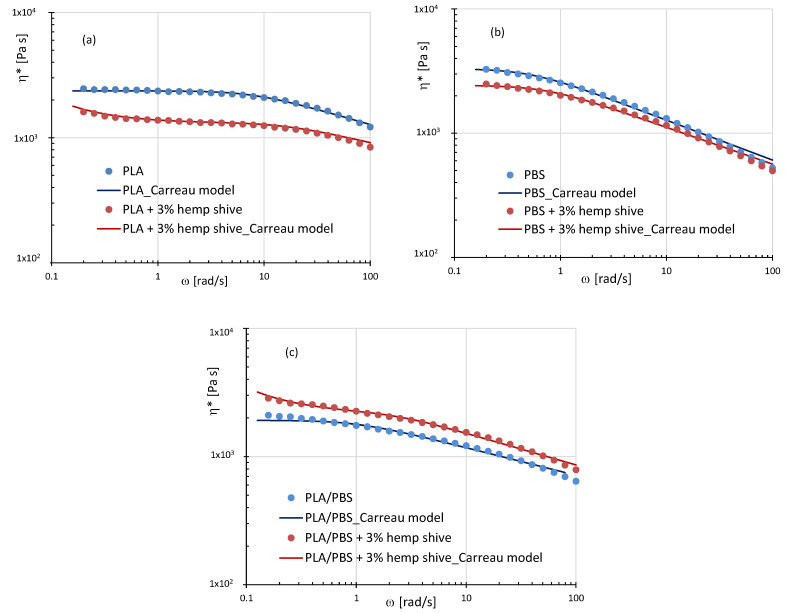
Experimental viscosity data and corresponding fits using the Carreau model (solid lines) for: (**a**) neat PLA, (**b**) PBS, and (**c**) PLA/PBS (50/50 wt%) systems, as well as those reinforced with 3 wt% hemp shives.

Since the validity of the Cox–Merz rule has been demonstrated in the literature [32] for systems similar to the ones investigated in this study, dynamic rheological data were fitted using both the traditional Carreau model (Equation (1)) and a modified version (Equation (2)), the latter accounting for the occurrence of yield stress in the composite systems.

(1) $$ƞ\left(\omega \right)=ƞo{\left[1+{\left(\lambda \omega \right)}^{2}\right]}^{\frac{n-1}{n}}$$

(2) $$ƞ\left(\omega \right)=ƞo{\left[1+{\left(\lambda \omega \right)}^{2}\right]}^{\frac{n-1}{n}}+\frac{\sigma_{0}}{\omega }$$

where ƞ_0_ represents the zero-shear viscosity, *λ* is the characteristic relaxation time, *n* is the power-law index describing the slope of the viscosity curve in the shear-thinning region, and *σ*_0_ is the yield stress. In the Appendix A, Figure A2 shows the experimental η* values fitted using the Carreau models (solid lines) for PLA-, PBS-, and PLA/PBS-based systems, both neat and containing 3 wt% of hemp shives. The corresponding model parameters for all tested materials are reported in Table 5.

Analysis of the tabulated data reveals that the relaxation times (*λ*) consistently decrease upon the addition of the filler, regardless of the polymer matrix. On average, the composites exhibit relaxation times nearly halved compared to those of the corresponding neat polymers. The power-law index (*n*) decreases in neat systems containing PBS, reflecting its inherently more pronounced shear-thinning behavior compared to PLA. In contrast, the incorporation of hemp slightly increases *n* values in both PLA- and PBS-based matrices, whereas an opposite trend is observed for the filled blends. Yield stress was evaluated exclusively for PLA-based composites, whose viscosity profiles were fitted using the modified Carreau model. Notably, yield stress remained essentially unchanged with increasing filler content.

**Figure 8:**
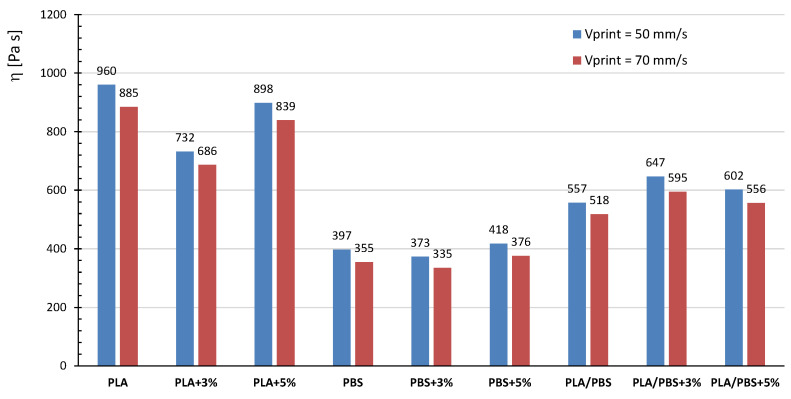
Viscosity values of PBS-, PLA- and PLA/PBS (50/50 wt%)-based systems at nozzle shear rates corresponding to printing speeds of 50 and 70 mm/s.

These models were therefore employed to extrapolate the viscosity values of the different systems at the shear rates estimated to occur within the nozzle during extrusion (Figure 8). The obtained results show that PBS-based materials exhibit the lowest viscosity values within the printer nozzle—in agreement with their more pronounced shear-thinning behavior—compared to PLA and PLA/PBS systems. Moreover, at the tested printing speeds, the viscosity of PBS-based systems does not change significantly with increasing filler content, whereas incorporating 3 wt% of hemp shive into PLA leads to a marked reduction in viscosity. In contrast, the filler addition inside PLA/PBS matrix results in a slight increase in viscosity.

In summary, all the analyzed systems exhibit viscosity values within the range of 100 to 1000 Pa·s, which has been previously identified in the literature as favorable for ensuring adequate flowability of polymer melts through the nozzle during FDM printing [12,34]. However, with the awareness that the absolute reliability of these extrapolated viscosities is limited, in future work, tests by means of capillary rheometer will be conducted to directly measure viscosity values in the shear rate range relevant to the FDM process.

Upon exiting the printer nozzle, the elastic recovery of stretched polymer chains may induce die-swell, which can affect the geometry of the printed objects. This phenomenon should be carefully controlled by adjusting the material’s formulation and/or the processing conditions. The goal is to achieve a certain degree of overlap between printed layers, resulting in a more compact structure—which is beneficial for the final mechanical performance—while still preserving the intended dimensions of the printed sample.

**Figure 9:**
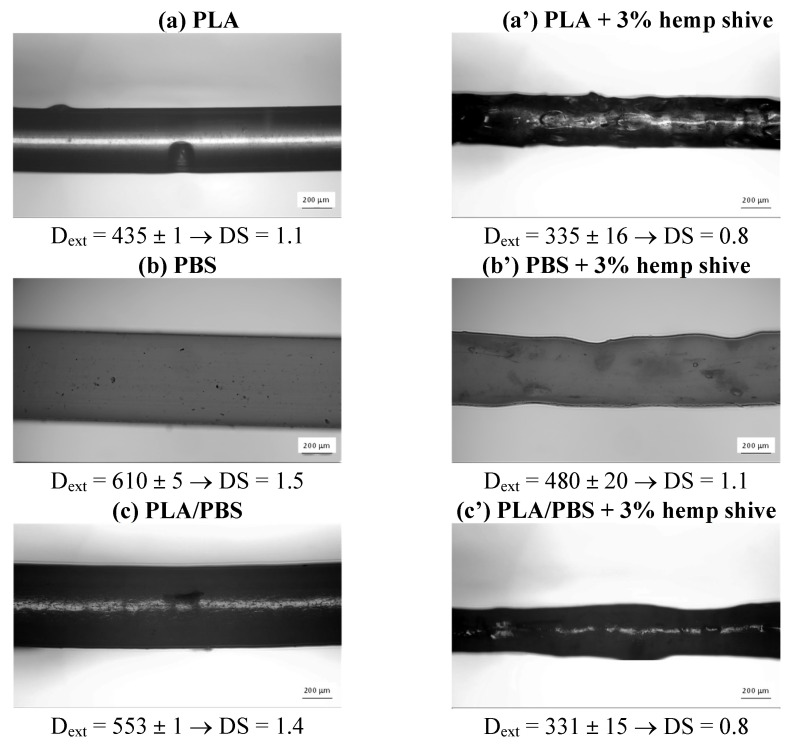
Images of the extruded filaments and die-swell values for: (**a**) neat PLA, (**b**) PBS, (**c**) the PLA/PBS (50/50 wt%) blend and the (**a’**–**c’**) corresponding composite at 3 wt% of hemp shive.

An approximative evaluation of die-swell behavior was carried out by extruding filaments of neat PLA, PBS, and their 50/50 blend, both unfilled and reinforced with 3 wt% hemp shives. The extrudates were collected directly at the nozzle exit and analyzed via optical microscopy (Figure 9). The corresponding die-swell (DS) values was calculated as the ratio between the diameter of the extruded filament (D_ext_) and the nozzle diameter (D_nozzle_).

Among the neat materials, PLA exhibits relatively low die-swell (DS = 1.1), while PBS and the PLA/PBS blend show a more pronounced expansion (DS = 1.5 and DS = 1.4, respectively). Conversely, the addition of hemp shive results in a reduction in die-swell for all the investigated systems, particularly pronounced for the PLA/PBS blend, with the value decreasing to DS = 0.8.

As well established in the literature [48], the extent of the die-swell phenomenon is strongly influenced by the molecular characteristics and/or morphology of the material, which affect its elastic response and, in turn, its characteristic relaxation times. In particular, PBS is characterized by longer relaxation times than PLA (Table 5), thus extending the overall relaxation behavior of PLA/PBS blend. Moreover, since the blend is immiscible (Figure 2), the interfacial elasticity, arising from the phases’ boundaries, further amplifies the die-swell effect [49].

The reduction in die-swell, due to the addition of hemp shive, can be attributed to two main mechanisms. Firstly, the filler exhibits a plasticizing effect (Figure 5 and Figure 6), resulting in reduced relaxation times during melt flow within the nozzle. Secondly, at the nozzle exit—where shear rates significantly decrease—the fillers’ microstructural network imposes a physical constraint on the polymer chains, thereby limiting their elastic recoil and further suppressing swelling behavior. Furthermore, hemp shives improve polymer phases’ compatibility within the PLA/PBS blend, as evidenced by the FTIR spectra in Figure 3. This results in a more homogeneous system and, from a rheological perspective, contributes to a further reduction in the swelling phenomenon for the filled blend [49].

### 3.3. Effect of Hemp Shive and 3D Printing Parameters on Morphology and Performances of Biocomposites

In the second phase of this study, printing tests were carried out by varying two key process parameters: printing speed and nozzle temperature. The printed specimens were evaluated for surface and dimensional accuracy using optical microscopy and subsequently characterized mechanically through flexural and tensile tests.

As predictable by the results of the rheological characterization, all the investigated systems were successfully and smoothly extruded through the printer nozzle, obtaining uniform and consistent printed objects. Moreover, no nozzle clogging occurred during the 3D printing of the composites. Since the average filler size was approximately half the nozzle diameter, this indirectly suggests a good distribution of the hemp shive throughout all the formulations. Only in the case of PBS-based systems—both the neat polymer and the composites with hemp shive—did a problem occur during the deposition phase. Their detachment from the build platform occurred, likely due to volumetric shrinkage and the development of high residual stresses during cooling of these specimens. This behavior can be attributed to the faster crystallization kinetics of PBS compared to PLA, in agreement with previous findings reported in the literature for neat PBS [45]. The incorporation of hemp shive powder did not appear to significantly alter the crystallization behavior of the polymer matrix in a way that improves the printability of PBS-based composites. As this issue lies beyond the scope of the present study, it will be addressed in greater detail in future investigations.

**Figure 10:**
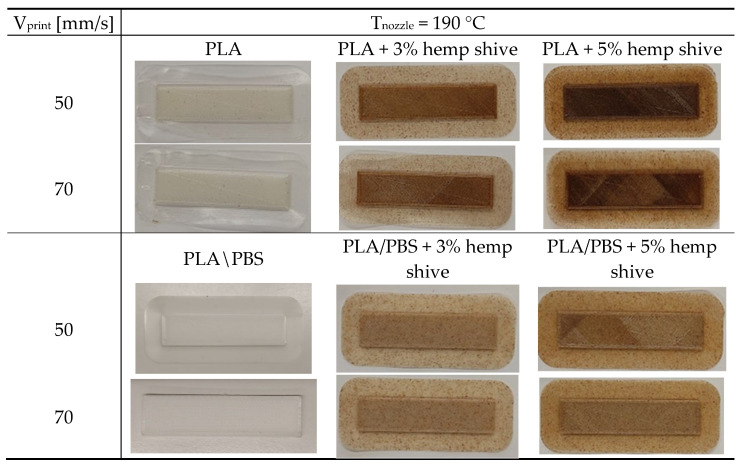
Photos of PLA and PLA/PBS (50/50 wt%) systems produced at 190 °C and printing speed of 50 and 70 mm/s.

Figure 10 presents the images of PLA- and PLA/PBS-based samples, both neat and filled with hemp shive, produced at nozzle temperature of 190 °C and at printing speeds of 50 and 70 mm/s.

**Figure 11:**
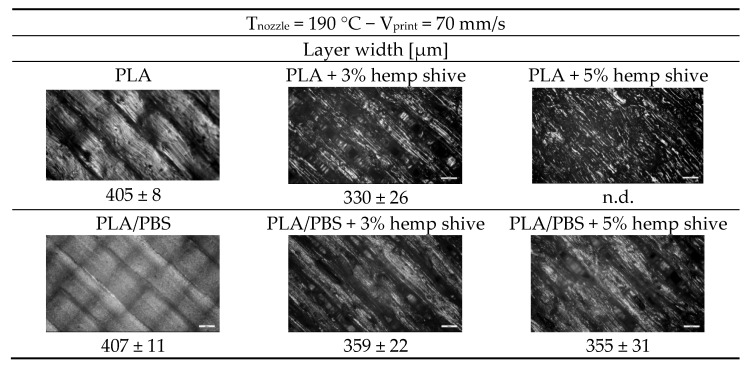
Optical microscopy images of PLA and PLA/PBS (50/50 wt%) systems printed at 190 °C and 70 mm/s, with the corresponding measurements of layer width.

To the naked eye, the samples printed at 70 mm/s display dimensional uniformity similar to those printed at the lower speed, with the added benefit of reduced printing times. The filled systems exhibit increased surface roughness and irregularities compared to their neat counterparts, with these effects becoming more pronounced at higher hemp shive contents. Additionally, increasing the filler loading results in a visible darkening of the printed samples. The presence of hemp shive particularly impacted the quality appearance of PLA-based composites. To enable a more detailed evaluation of surface quality and dimensional accuracy, optical microscopy analysis was conducted, and the layer width was measured for the samples printed at 70 mm/s (Figure 11).

As expected, the unfilled samples exhibited smoother and more regular layer surfaces compared to their hemp-filled counterparts. Moreover, the neat matrices showed a more compact morphology, particularly the blend, in which individual layers were barely distinguishable. Specifically, for the neat samples, the measured layer width was approximately 400 µm, matching the nozzle diameter. In contrast, the hemp-filled systems displayed average layer width values lower than the nominal dimensions. The observed morphologies can be consistently associated with the extent of die-swell occurring at the nozzle exit, which varied across the different systems analyzed (Figure 9).

The subsequent phase of this study focused on evaluating the effect of nozzle temperature on the morphology and properties of the printed specimens. This analysis was limited to the neat systems, as elevated printing temperatures—while potentially enhancing macromolecular interdiffusion between adjacent layers and, thus, improving interlayer adhesion and the overall structural performance of the printed parts—pose a risk to the thermal stability of the filler. Specifically, thermogravimetric analysis of the hemp shive (Figure 1) indicated significant thermal degradation occurring at temperatures exceeding 200 °C.

**Figure 12:**
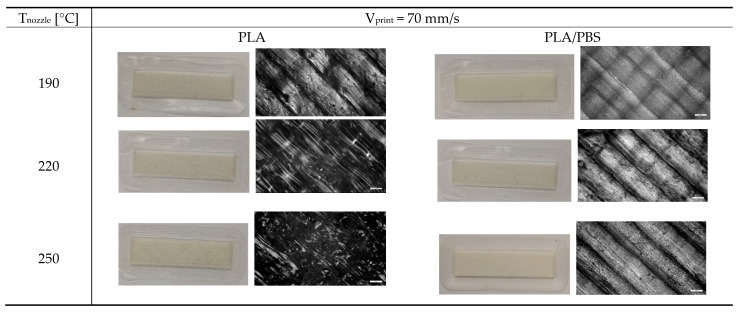
Photos and optical microscopy images of neat PLA and PLA/PBS (50/50 wt%) samples printed at 70 mm/s and different nozzle temperatures.

Figure 12 compares the photographs of PLA and PLA/PBS samples printed at 190, 220, and 250 °C, as well as their corresponding optical microscopy images.

PLA specimens show some surface irregularities as the nozzle temperature increased, while PLA/PBS samples do not exhibit signs of thermal degradation, coherently with the higher thermal stability of PBS compared to PLA [43].

**Figure 13:**
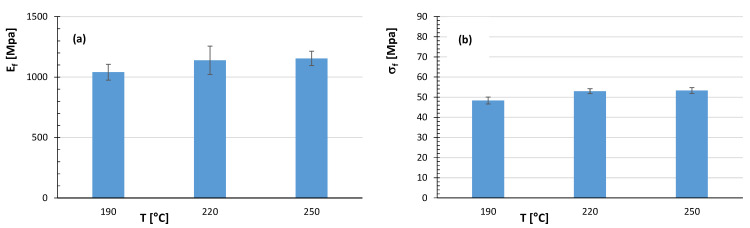
Values of (**a**) flexural modulus and (**b**) flexural strength for PLA/PBS (50/50 wt%) samples printed at 70 mm/s and different nozzle temperatures.

The flexural properties of the PLA/PBS blend as a function of nozzle temperature (190, 220, and 250 °C) are reported in Figure 13. In particular, a gradual but modest improvement in both flexural modulus and flexural strength can be observed for PLA/PBS blend by increasing the nozzle temperature.

It is important to note that interlayer adhesion in 3D-printed parts is influenced not only by the enhanced macromolecular diffusion at elevated temperatures, but also by the material’s crystallization kinetics, which play a crucial role in determining the strength of the interfaces [50]. So, further investigations need to be performed in order to obtain a deeper understanding of the materials’ performance by increasing the printing temperature.

In the final stage of this study, the effect of hemp shive content on the mechanical properties of both PLA and PLA/PBS blend was evaluated. For this purpose, all specimens were printed at 70 mm/s and a nozzle temperature of 190 °C, as it was demonstrated that these printing parameters allow for the best compromise between the samples’ quality appearance/performances and time/energy consumption issues.

As shown in Table 6, the addition of hemp shive to PLA matrix leads to an increase in stiffness.

Specifically, the composite containing 3 wt% filler exhibited a ~30% increase in flexural modulus compared to neat PLA. However, a gradual—though modest—reduction in flexural strength was observed with increasing filler content, evidencing limited interfacial adhesion between PLA and hemp shive powder. Moreover, both the neat PLA and the filled samples fractured before reaching the maximum deflection limit, due to the inherent brittleness of these materials. Test results for PLA/PBS-based systems (Table 6) showed an overall decrease in both flexural modulus and strength compared to PLA-based samples, since the PBS phase is less stiff and strong than polylactic acid. As observed for PLA-based composites, the addition of hemp shive to the PLA/PBS blend resulted in a slight increase in stiffness. A moderate reduction in flexural strength was observed only at the highest filler content. However, unlike PLA-based systems, none of PLA/PBS specimens fractured at the maximum imposed deflection during testing, highlighting an improvement in ductility due to the presence of PBS phase.

To further investigate the effect of the filler on the fracture properties of 3D-printed PLA and PLA/PBS specimens, tensile tests were performed on both the neat materials and the composites with 3 wt% of hemp shive. Table 7 reports the stress and strain values at the yield point (σ_y_, ε_y_) and at break (σ_b_, ε_b_) for the tested specimens.

The neat PLA/PBS blend exhibits an 8.6-fold increase in the elongation at break compared to PLA, with only a 38% reduction in tensile strength. The addition of 3 wt% of hemp shive leads to increased brittleness for both the PLA and PLA/PBS systems with respect to their unfilled counterparts. Nevertheless, the PLA/PBS-based composite shows an elongation at break nearly three times higher than that of the corresponding PLA-based system. To enable a comprehensive interpretation of the results, the mechanical performance of the composites must also be correlated with their morphology, which was found to be less compact compared to that of the corresponding unfilled systems (Figure 12).

Further investigations—such as scanning electron microscopy (SEM) analyses and density measurements—would allow for a more comprehensive understanding of the relationship between void fraction and the mechanical strength of the composites. These will be considered in future work, together with the optimization of printing parameters—for instance, by introducing an appropriately selected overlap value—to minimize void content and enhance the overall mechanical performance of hemp shive composites.

## 4. Conclusions

This work explored the 3D-FDM processability and mechanical performance of biocomposites based on PLA, PBS and a 50/50 wt% PLA/PBS blend reinforced with hemp shive powder, a lignocellulosic by-product of hemp fiber processing. The rheological analysis highlighted how the PLA/PBS blend effectively combines the advantageous features of both polymers, showing enhanced shear-thinning behavior and improved viscous characteristics for extrusion-based additive manufacturing.

The structuring effect of hemp shive inside the polymer matrices significantly reduced the material swelling at the nozzle exit, resulting in less compact printed structures, ultimately impairing the final performances of the composites.

Optimal printing conditions were achieved at a nozzle temperature of 190 °C and a printing speed of 70 mm/s, offering a good balance between print quality, mechanical properties, and energy/time efficiency. From a mechanical perspective, adding 3 wt% of hemp shives to PLA increased stiffness by approximately 30%, though it also reduced ductility. In contrast, the PLA/PBS blend demonstrated a remarkable 8.6-fold increase in elongation at break compared to neat PLA. Even after hemp shive reinforcement, the blend-based composite maintained ductility values nearly three times higher than those of its PLA-based counterpart.

Overall, the PLA/PBS blend reinforced with hemp shive presents a promising and sustainable formulation for FDM applications, delivering a well-balanced compromise between processability, print quality, mechanical performance and environmental impact.

## Figures and Tables

**Table 1:** Main characteristics of PLA and PBS resins.

Property	Test Method	Unit	PBS FZ91PM	PLA 4032D
Density	ISO 1183	g/cm^3^	1.26	1.24
Melting temperature	DSC	°C	115	170
Glass transition temperature	DSC	°C	−32	60

**Table 2:** Processing parameters used for composites’ production.

Sample	T_1_ (°C)	T_2_ = T_7_ (°C)	T_8_ = T_die_ (°C)
PBS-based composites	150	165	165
PLA-based composites PLA/PBS-based composites	170	190	180

**Table 3:** Processing parameters used for filaments’ production.

Sample	T_1_ (°C)	T_2_ = T_4_ (°C)	Fan Speed [%]
PBS	150	165	100
PBS-based composites	170	170	75
PLA PLA-based composites	200	200	65
PLA/PBS PLA/PBS-based composites	180	180	75

**Table 4:** FTIR peaks assignments for PLA, PBS and hemp shive.

PLA	
Wavenumber [cm^−1^]	Assignment
1746	Stretching CO
1452	Asymmetric bending CH_3_
1358–1382	Symmetric bending CH_3_
1266	Bending Chand stretching COC
1180	Asymmetric rocking CH_3_
1127	Asymmetric rocking CH_3_
1080	Symmetric stretching COC
1042	Symmetric stretching C-CH_3_
**PBS**	
Wavenumber [cm^−1^]	Assignment
1712	Stretching CO
1469–1308	Deformation C–H in CH_2_
1149	Asymmetric stretching CO in COC
1044	Symmetric stretching C–O in COC
**Hemp shive**	
Wavenumber [cm^−1^]	Assignment
2915	Stretching CH (cellulose, hemicellulose)
2896	Symmetric stretching (polysaccharides)
1732	Stretching CO (hemicellulose)
1502	Aromatic symmetrical stretching (lignin)
1421	Bending CH_2_ (cellulose)
1370	Bending CH_2_ (cellulose)
1318	In plane bending OH (cellulose)
1157	Asymmetric oxygen stretching COC (cellulose)
1032	Ring and side group vibration C–C, C–OH, C–H (hemicellulose, pectin)

**Table 5:** Model parameters of the Carreau equations for neat PLA, PBS, and PLA/PBS (50/50 wt%) blend, as well as their composites loaded with 3 and 5 wt% hemp shives.

	η_0_ [Pa·s]	*λ* [s]	*n*	*σ*_0_ [Pa]	R
PLA	2362	0.125	0.76	-	0.991
PLA + 3% hemp shive	1307	0.065	0.81	75	0.987
PLA + 5% hemp shive	1679	0.069	0.80	88	0.997
PBS	2961	1.247	0.66	-	0.997
PBS + 3% hemp shive	2091	0.716	0.68	-	0.999
PBS + 5% hemp shive	2051	0.532	0.69	-	0.998
PLA/PBS	1916	0.960	0.78	-	0.992
PLA/PBS + 3% hemp shive	2175	0.431	0.75	-	0.995
PLA/PBS + 5% hemp shive	2062	0.437	0.77	-	0.996

**Table 6:** Flexural mechanical properties of PLA and PLA/PBS (50/50 wt%) systems.

	T_nozzle_ = 190 °C − V_print_ = 70 mm/s	
	Flexural Modulus E_f_ [MPa]	Flexural Strength σ_f_ [MPa]
PLA	1680 ± 60	71 ± 4
PLA + 3% hemp shive	2210 ± 230	62 ± 13
PLA + 5% hemp shive	2000 ± 300	58 ± 12
PLA/PBS	1050 ± 60	48 ± 2
PLA/PBS + 3% hemp shive	1070 ± 70	47 ± 3
PLA/PBS + 5% hemp shive	1150 ± 70	38 ± 5

**Table 7:** Tensile mechanical properties of PLA and PLA/PBS (50/50 wt%) systems.

Sample	ε_Y_ [%]	σ_Y_ [MPa]	ε_b_ [%]	σ_b_ [MPa]
PLA	11.3 ± 0.3	44.5 ± 1.5	14.7 ± 1.6	40.0 ± 2.3
PLA + 3% hemp shive	9.6 ± 0.7	38.3 ± 3.3	9.6 ± 0.7	38.3 ± 3.3
PLA/PBS	15.3 ± 1.2	42.3 ± 0.2	126.9 ± 24.4	24.6 ± 1.4
PLA/PBS + 3% hemp shive	16.2 ± 1.2	34.3 ± 2.3	27.4 ± 5.1	24.5 ± 4.1

## Data Availability

Data are contained within this article.

## Abbreviations

The following abbreviations are used in this manuscript:

AM	Additive Manufacturing
FDM	Fused Deposition Modeling
CAD	Computer-Aided Design

## Appendix A

Considering that the printer was equipped with a nozzle of 0.4 mm in diameter and that a layer height of 0.1 mm was set, the shear rates inside the nozzle were estimated to be approximately 320 s^−1^ and 450 s^−1^ at the tested printing speeds of 50 mm/s and 70 mm/s, respectively.
